# Unusual ferromagnetism enhancement in ferromagnetically optimal manganite La_0.7−__*y*_Ca_0.3+__*y*_Mn_1−__*y*_Ru*_y_*O_3_ (0≤*y*<0.3): the role of Mn-Ru *t_2g_* super-exchange

**DOI:** 10.1038/srep09922

**Published:** 2015-04-24

**Authors:** M. F. Liu, Z. Z. Du, Y. L. Xie, X. Li, Z. B. Yan, J. –M. Liu

**Affiliations:** 1 Laboratory of Solid State Microstructures and Innovative Center of Advanced Microstructures, Nanjing University, Nanjing 210093, China; 2 Institute for Advanced Materials and Laboratory of Quantum Engineering and Materials, South China Normal University, Guangzhou 510006, China

## Abstract

The *e_g_*-orbital double-exchange mechanism as the core of physics of colossal magnetoresistance (CMR) manganites is well known, which usually covers up the role of super-exchange at the *t_2g_*-orbitals. The role of the double-exchange mechanism is maximized in La_0.7_Ca_0.3_MnO_3_, leading to the concurrent metal-insulator transition and ferromagnetic transition as well as CMR effect. In this work, by a set of synchronous Ru-substitution and Ca-substitution experiments on La_0.7–*y*_Ca_0.3+*y*_Mn_1–*y*_Ru*_y_*O_3_, we demonstrate that the optimal ferromagnetism in La_0.7_Ca_0.3_MnO_3_ can be further enhanced. It is also found that the metal-insulator transition and magnetic transition can be separately modulated. By well-designed experimental schemes with which the Mn^3+^-Mn^4+^ double-exchange is damaged as weakly as possible, it is revealed that this ferromagnetism enhancement is attributed to the Mn-Ru *t_2g_* ferromagnetic super-exchange. The present work allows a platform on which the electro-transport and magnetism of rare-earth manganites can be controlled by means of the *t_2g_*-orbital physics of strongly correlated transition metal oxides.

In the past more than twenty years, alkaline earth doped manganites R_1-*x*_D*_x_*MnO_3_ (abbreviated as manganites hereafter), where R is the rare-earth ion and D is the alkaline earth ion, have received extensive attention as a representative class of strongly correlated electron systems^1^,^2^,^3^. Nowadays, it is believed that the physics of manganites is well understood and thus no longer the hot topic. Three major milestones for the physics of manganites are generally believed. First, the concept of double-exchange (DE) for the *e_g_* electrons and super-exchange (SE) for the *t_2g_* electrons between two neighboring Mn ions bridged by one O^2-^ ion (one Mn-O-Mn chain unit) has been adopted to explain the ferromagnetic (FM) transition plus the metal-insulator transition (MIT) as well as colossal magnetoresistance (CMR) effect^4^,^5^,^6^,^7^. The two types of interactions are schematically highlighted in Fig. 1(a)∼(c) for a Mn-O-Mn unit, where O^2−^ ion is ignored for the sake of simplification^6^,^7^. This model is discussed on the assumption that the Mn-O-Mn bond angle is not far from 180°, while in many cases it is indeed far from 180° due to the lattice distortion including the Jahn-Teller distortion. For instance, the Mn-O-Mn bond angles for La_0.7_Sr_0.3_MnO_3_ and La_0.7_Ca_0.3_MnO_3_, which have almost the strongest FM tendency in La_1-x_Sr_x_MnO_3_ (LSMO) and La_1-x_Ca_x_MnO_3_ (LCMO) families, are 166.1° and 160.3°^8^,^9^. Second, the scenario of electronic phase separation (EPS) due to prominent quenched disorder in some manganites has been demonstrated to be responsible for the huge variations of magneto- and electro-transport properties and for remarkable dynamic characteristics of these properties. When the EPS effect is significant, these variations are somewhat induced by the field driven transitions and no longer intimately relevant with the DE mechanism^7^,^10^,^11^,^12^,^13^,^14^. Third, a series of emergent quantum phase transitions as consequences of strong correlations between charge, spin, orbit, and lattice degrees of freedom have been identified^7^,^11^. These milestones comprise the mainstream of the physics of manganites, and also feature the complexity of strongly correlated electron physics^1^,^2^,^3^,^4^,^5^,^6^,^7^,^8^,^9^,^10^,^11^,^12^.

The physics of manganites is dominated by the DE process and the competition among those degrees of freedom. This DE process generates two phenomena: MIT and FM transition^4^,^15^,^16^, which may not be necessarily correlated. Instead, the *t_2g_*-orbital super-exchange is seemingly less pronounced in determining these emergent phenomena. We take LCMO family as an example, noting that the EPS in LCMO is non-negligible but insignificant^7^. To some extent, the Jahn-Teller effect in LCMO is important but not so sensitive to intrinsic substitutions and external stimuli too^4^, and therefore will not be considered here for convenience of discussion. Upon the Ca^2+^ partial substitution of La^3+^, the as-generated Mn^4+^ ions coexist with Mn^3+^ ions, giving the Mn^4+^/Mn^3+^ ratio *η* = *x*/1-*x*. The FM transition point *T_C_* reaches the maximal *T_C_*~265K at *x*~0.3, at which the Mn^4+^ charge density *d*_4+_ is ~0.3 and the Mn^3+^ charge density *d*_3+_ is ~0.7. Further increasing of *x* leads to spatially ordered Mn^4+^:Mn^3+^ charge-ordering (CO) and even orbital-ordering (OO) sequence^17^,^18^,^19^,^20^,^21^,^22^,^23^, which on the other hand favors the antiferromagnetic (AFM) insulating transitions at *x*~0.5 and above. Therefore, the optimal ferromagnetism and best metallic conductivity in LCMO appears at *x* = 0.3.

For LCMO at *x* = 0.3, the role of the *t_2g_* super-exchange seems to be completely screened in terms of the electro-transport and magnetism, or their impact remains far from significant^24^,^25^. This issue has rarely been questioned in comparison with R_1-*x*_D_x_CoO_3_ where the *t_2g_* exchange becomes important. For example, how do the electro-transport and magnetism respond if the *t_2g_* super-exchange from locally antiferromagnetic (AFM) interaction into locally FM interaction is modulated? In this sense, any approach to enhance the *T_C_* via modulating the local *t_2g_* super-exchange would be of interest and highly appreciated not only for practical applications but also for uncovering additional physics of manganites.

In fact, a number of substitution experiments on LCMO by replacing Mn with other transition metal species, e.g. La_1−*x*_Ca*_x_*Mn_1−*y*_Me*_y_*O_3_, have been carried out^26^,^27^,^28^. Here Me = Fe, Cr, Co, etc and 4*d* ions in some cases. In most cases the substitutions introduce remarkable quenched disorder and interaction frustrations. For *x* = 0.3, these substitutions will lower the FM transition point *T_C_* by weakening the double-exchange, and so far no many reports on enhancing the *T_C_* in this manganite by means of the Mn-site substitution are available^28^. These experiments may not be unambiguous to uncover the role of the *t_2g_* super-exchange without inducing other complexities. First, the 3*d* ions may have similar *e_g_* levels as those of Mn ion, and thus additional DE effect can’t be excluded. In many cases these effects are much more significant than the *t_2g_*-orbital super-exchange^29^,^30^. So far intention to outshoot the role of the *t_2g_* super-exchange is unsatisfactory. Second, these 3*d* ions usually have more than one valence state and the substitutions may lead to unexpected variation of Mn valence states and oxygen vacancies^31^. The DE process will be seriously disturbed. Third, these substitutions will induce significant quenched disorder and thus EPS^12^,^14^.

A handle of these problems is challenging. A promising approach needs to satisfy the following requirements in the low substitution levels. (1) The density of Mn^4+^ ions as minor chargers should be maintained, so that the density of empty *e_g_* orbitals allowing for electron hopping will not be much disturbed. (2) At best the DE process between Mn ions and substituting ions is prohibited but the *t_2g_* super-exchange can be modulated as much as possible. (3) For most cases the *t_2g_* super-exchanges are AFM. If one is able to introduce an FM super-exchange ingredient at the *t_2g_* orbitals without affecting much the double-exchange, the impact of the *t_2g_* super-exchange definitely deserves for investigation. Along this line, a substitution of Mn by 4*d* Ru^4+^ is highly appreciated^26^,^27^. Before going to details of Ru-substitution in LCMO, we have an outline of Ru-substitution in manganites including the end DMnO_3_ systems (*x* = 1). In spite of the scattering data, all conclusions state that the Ru-substitution enhances the ferromagnetism. However no detailed discussion on the role of *t_2g_* super-exchange has been addressed regarding this ferromagnetism enhancement. We again take LCMO at *x* = 0.3 as an example for illustration of the reasons. Here the Mn-O-Mn bond angle is not so far from 180°^24^,^25^. This allows a relatively simple scheme of interactions. We consider a neighboring Mn-Ru pair bridged with an O^2-^ ion. The two cases are schematically drawn in Fig. 1(d)∼ (e) where the *e_g_*- and *t_2g_*-orbital structures between neighboring Mn^3+^-Ru^4+^ and Mn^4+^-Ru^4+^ pairs are plotted. At the same time, the *d*-orbital structure for a Ru^4+^-Ru^4+^ pair is shown in Fig. 1(f) for reference, where the *t_2g_*-orbital electron hopping is allowed, as found in SrRuO_3_ and CaRuO_3_^32–34^. It is noted that Ru ion prefers the Ru^4+^ valence although other valence states are more or less claimed^34^.

Several aspects of possible physics upon the Ru^4+^ substitution need to be addressed. First, the *e_g_*-orbitals of Ru^4+^ ion are sufficiently higher than those of Mn ion^35^. This excludes the *e_g_*-orbital double-exchange between Mn^3+^-Ru^4+^ pair, as indicated in Fig. 1(d). Second, electron hopping between Ru^4+^ *t_2g_*-orbitals and Mn *e_g_*-orbitals can be questioned too, as indicated in Fig. 1(d) and (e). Instead, the Mn-Ru *t_2g_* super-exchange should be considered. In a good approximation, the *t_2g_*-orbital hybridization and coupling between Mn^3+^-Ru^4+^ and Mn^4+^-Ru^4+^ pairs lead to the FM interaction, which is a critical ingredient of physics we need to consider in this work. In fact, several earlier experiments did reveal the Mn-Ru FM interaction^26^,^27^,^29^,^30^,^34^, which should be but has not yet been ascribed to this *t_2g_*-orbital FM super-exchange. Third, if *x* is fixed, the Ru-substitution will reduce the *d*_4+_, which certainly damages seriously the Mn^3+^-Mn^4+^ DE sequence and covers up the role of the *t_2g_* super-exchange. A better strategy is to ensure the Mn^3+^-Mn^4+^ DE sequence as disturbed as weak by the Ru substitution. This can be realized in La_0.7-*y*_Ca_0.3+*y*_Mn_1-*y*_Ru*_y_*O_3_ (LCMRO), as long as the *d*_3+_ remains sufficiently high, e.g. at *y*<0.3. It will be shown below that the evaluated Mn-O-Ru bond angle from the structural fitting is ~160°, similar to the Mn-O-Mn bond angle of La_0.7_Ca_0.3_MnO_3_, suggesting that the overlapping between the Mn *e_g_* level and Ru *t_2g_* level is weak if any and the scenario shown in Fig. 1(d) and (e) is reasonable. By this scheme, the influence of the Mn^3+/4+^-Ru^4+^ *t_2g_* FM super-exchange can be checked by characterizing the magnetism and electro-transport of LCMRO. This is the main motivation of the present work.

Here it should also be mentioned that such a Ru substitution with synchronous Ca substitution in LCMRO will not change the lattice structure much, considering that the ionic sizes of La^3+^, Ca^2+^, Mn^3+^, Mn^4+^, and Ru^4+^ in the 9-coordination frame, are 1.03, 1.00, 0.64, 0.53, and 0.62Å, respectively^36^. Obviously, given roughly constant *d*_4+_, any reduction of the *d*_3+_ will damage more or less the electrical conductivity. The quenched disorder and thus the EPS induced by the Ru substitution will be inevitable but negligible, since nearly no thermal hysteresis has been observed for both the magnetization and electrical resistivity measurements. To this stage, we have proposed a scheme for uncovering the effect of the Mn^3+/4+^-Ru^4+^ *t_2g_* FM super-exchange on the electro-transport and magnetism in LCMRO, and the detailed data are presented below.

## Results

### Structural characterizations

Both LCMO and CaRuO_3_ exhibit orthorhombic structure with space group *Pnma*^24^,^32^. Due to similar lattice structures, ionic occupations, and one-to-one corresponding ionic sizes, no serious change in lattice symmetry for the Ru substitution of Mn with synchronous Ca occupation at La site is expected. The measured *θ*−2*θ* XRD spectra for a series of samples are presented in Fig. 2(a). The locally amplified reflections around 2*θ* = 46° ~48° and 68°~70° are presented in Fig. 2(b) and (c), respectively, showing gradually rightward shifting. This is reasonable considering the ionic size mismatch^36^. In addition, the lattice constants (*a*, *b*, *c*) as a function of *y* respectively, as evaluated by means of the Rietveld refinement processing, are plotted in Fig. 2(d)∼(f). All the three constants decrease slightly with increasing *y*, while the unit cell volume *V* shows a linear dependence on *y*, satisfying the Vegard’s law^37^. This implies that the Ru valence remains to be identical in all the samples, which otherwise would result in an identifiable deviation of the *V*(*y*) from the Vegard’s law. Here it should be mentioned that the Rietveld refinements confirm the cation ratios for all the samples and one example is given in the Supplementary information.

Further evidence is given by the XPS identification. Fig. 3 presents the XPS data for five samples. In Fig. 3(a) are shown the local non-shifting Ru peaks even when *y* is as high as 0.5. The XPS spectra covering the 2*p*_1/2_ and 2*p*_3/2_ peaks from an overlap of Mn^3+^ and Mn^4+^ are plotted in Fig. 3(b). The overall gradual shifting of the two broad peaks towards the high-energy side with increasing *y* suggests that the Mn^3+^ density is gradually lowered. This is strong evidence supporting the gradually lowered Mn^3+^ density (*d*_3+_). A rough estimation of the Mn^4+^/Mn^3+^ ratios for several samples in spite of relatively big errors of XPS gives similar results and one example is given in Fig. 3(c) for *y* = 0.25, where the best fittings using the Gaussian modes to the 2*p*_1/2_ and 2*p*_3/2_ of Mn^4+^ and Mn^3+^ are illustrated. The evaluated Mn^4+^/Mn^3+^ ratio is ~0.336 with an uncertainty of ±5%~10%, confirming that the *d*_4+_ remains to be ~0.3, independent of *y*.

Surely, the precision of the present XPS data may not be sufficient for excluding the existence of tiny amount of Ru^3+^ or Ru^5+^. However, given the fact that the probed Ru peaks don’t move over such a wide *y*-range from 0.05 to 0.5 but the Mn peaks shift remarkably (see Fig. 3(b)), one is allowed to suggest that the Ru^4+^ valence state is dominant in these samples even if tiny amount of Ru^3+^ or Ru^5+^ is available. In addition, earlier work^38^ did report the Ru^3+^/Ru^5+^ valent states in La_0.7_Sr_0.3_Mn_1−x_Ru_x_O_3_ where the La and Sr contents are constant. However, in our samples, the contents of La, Ca, Mn, and Ru all change synchronously so that the dominant Ru^4+^ state is favored for the charge neutrality. Furthermore, for the case of high *y* value (*y* = 0.5) where the Ru^3+^ state was argued to be favored^39^, no peak shift with respect to those samples with lower *y* can be seen, as shown in Fig. 3.

### Magnetic and electro-transport behaviors

In prior to discuss the magnetic and transport data, we check the possible EPS in our samples. It is known that well developed EPS in manganites is usually accompanied with remarkable low-*T* thermal hysteresis for both the *ρ*(*T*) and *M*(*T*) dependences if the measurement is performed in a cooling-warming cycle. We checked all the samples carefully and found no remarked hysteresis and the data for two samples are presented in the Supplementary information. It is seen that the difference between the cooling sequence and warming one is small, suggesting weak EPS if any in these samples.

In the low-*y* range, an immediate consequence is the gradually damaged electrical conductivity. A reduction of *d*_3+_ will certainly dilute the Mn^3+^-Mn^4+^ DE transport networks. To check this effect, we turn to the *ρ*(*T*) and *M*(*T*) in response to varying *y*, plotted in Fig. 4 for *y* up to 0.50. We mainly discuss the data in the low-*y* range (*y*<0.25). For LCMO shown in Fig. 4(a), the *ρ*(*T*) and *M*(*T*) dependences reproduce well the earlier data in literature. Upon decreasing *T*, the MIT at *T* = *T_1_* and FM transition at *T_C_* occur concurrently with *T_1_*∼*T_C_*~265K, featured by sharp *ρ*(*T*) peak and *M*(*T*) jump from paramagnetic state to FM state^24^. An additional weak bump of the *ρ*(*T*) dependence appears at a lower *T*∼*T_2_*~225K, while the overall dependence fits a typical metallic conduction. This bump feature has been well recorded in literature and its origin is believed to be the weak charge-ordering and consequent grain boundaries as weak links for electron transport, resulting in a weak resistivity peak at *T_2_*^40^. We are mainly concerned with the MIT and FM transition at *T_1_* and *T_C_*.

The observed consequences in the low-*y* range can be described from several aspects. First, the substitution does damage the metallic conduction below the MIT (*T_1_*), characterized by a rapid shifting of *T_2_* towards the low-*T* side and a slow overall up-rise of *ρ*(*T*) until *y*~0.10 where the MIT is nearly submerged. As *y*>0.1, the MIT feature is replaced by an inflexion point (*T_1_* in Fig. 4(d) and (e)), while the overall behavior is insulator-like. The maximal overall resistivity appears at *y*~0.20–0.25, beyond which the resistivity falls down rapidly. Second, different from the electro-transport, the FM transition point *T_C_* shifts toward the high-*T* side from 264K to 293K upon *y* increasing from 0.0 to 0.25. If the FM transition is induced by the Mn^3+^-Mn^4+^ DE process, the Ru-substitution should suppress instead of enhancing the FM transition. Beyond *y*~0.25, the *T_C_* begins to fall rapidly. Third, all the samples exhibit the typical FM transitions, noting that the measuring field is only 100 Oe.

In parallel to the MIT and FM transition, another consequence of the DE process is the CMR effect around the MIT^15^. The measured MR data at *H* = 6T are plotted in Fig. 5. The low-*y* samples do exhibit the CMR effect with the peak at *T*∼*T_MR_*. However, this effect becomes much less pronounced as *y*>0.15 and nearly disappears at *y*>0.20, implying that the Mn^3+^-Mn^4+^ double-exchange is suppressed at *y*>0.20. The observed MR behavior in the high-*y* range is attributed to the response of the EPS microstructure to increasing *H*^4^.

As a complimentary, one notices that the rapid decrease of *ρ* at *y*>~0.30 is attributed to the *t_2g_* electron hopping between neighboring Ru^4+^-Ru^4+^ pairs, which becomes remarkable at high *y*. It is known that CaRuO_3_ (SrRuO_3_ too) exhibits the metallic conduction due to this strong *t_2g_* electron hopping^32^. Also, the magnetic moment of Ru^4+^ is much smaller than that of Mn^4+^/Mn^3+35^, explaining the gradual reduction of *M* as *y*>0.30. On the other hand, the substitution will inevitably introduce quenched disorder, and the EPS-relevant phenomena become significant in the high-*y* range.

### Simple percolation model of Mn^3+^-Mn^4+^ double exchange transport

To understand the electrical transport in the low-*y* range, we start from a simple percolation model. Given *d*_4+_≡0.3, one has *d*_3+_ = 0.7-*y* and the total Mn^3+^+Mn^4+^ density (*d*_3+_+*d*_4+_) = 1.0-*y*, which both fall linearly with increasing *y*, following the formula La^3+^_0.7−*y*_Ca^2+^_0.3+*y*_Mn^3+^_0.7−*y*_Mn^4+^_0.3_Ru^4+^_y_O^2−^_3_. We introduce two reasonable assumptions. First, all the Mn^3+^, Mn^4+^, and Ru^4+^ are randomly distributed at B-sites. Second, electrons can’t hop along any path other than the Mn^3+^-Mn^4+^-Mn^3+^-Mn^4+^…channels via the DE mechanism. To simulate the electrical conduction, a huge number of resistor network samples on a 3D 100×100×100 cubic lattice with periodic boundary conditions are generated, where only the DE channels are conductive, as schematically shown in Fig. 6(a) by the olive color path.

The statistical averaging on these networks indicates a conduction percolation appearing at (*d*_3+_+*d*_4+_)~0.65, i.e. *y*~0.35, while the probability *p* for conduction as a function of (*d*_3+_+*d*_4+_) is plotted in Fig. 6(b). The percolation threshold (*y_c_*~0.35) is roughly consistent with experimentally observed *y*~0.25 at which the MIT point *T_1_* disappears, as seen in Fig. 4(e) and Fig. 4(f). In Fig. 6(b) are plotted the simulated results for *d*_4+_ = 0.25 and 0.20, suggesting remarkable dependence of *y_c_* on *d*_4+_. Considering the over-simplified DE conduction channels assumed in this toy model lattice as well the negative scattering effects of the less pronounced quenched disorder and EPS on the electron transport, our argument that the electrical conductivity is solely owing to the Mn^3+^-Mn^4+^ double-exchangeis reasonably confirmed, and no substantial contribution from the *t_2g_*-orbital electron hopping is believed.

## Discussion

### Origin for the unusual ferromagnetism enhancement

The above discussions confirm that the low Ru-substitution with synchronous Ca^2+^ substitution of La^3+^ suppresses the DE transport. The major unusual phenomenon is the enhanced FM transition *T_C_*. The dependence *T_C_*(*y*) is presented in Fig. 7(a), where the maximal MR temperature *T_MR_*, and the MR value at *T_MR_* under *H* = 6.0T, are plotted together for reference. The *ρ-y* data at *T* = 100K and 200K are presented in Fig. 7(b). As revealed earlier, the *T_C_*(*y*) increases linearly in the low-*y* range (*y*<0.25) and then falls rapidly at *y*>0.25, separating the substitution range into two regimes I and II. The *ρ*(*y*) in regime II is dominated by the Ru-Ru interaction, but we only deal with the behaviors in regime I. The *ρ*(*y*) at *T* = 100K and 200K show similar behavior: rapid increase in regime I and then fall in regime II. The *T_MR_*(*y*) dependence is nearly the same as the *T_C_*(*y*) while the *MR*(*y*) evidences a rapid fall in regime I, followed by a slow decaying in regime II. This slow decaying is most likely due to the resistivity variations associated with the less pronounced EPS.

**Figure 1:**
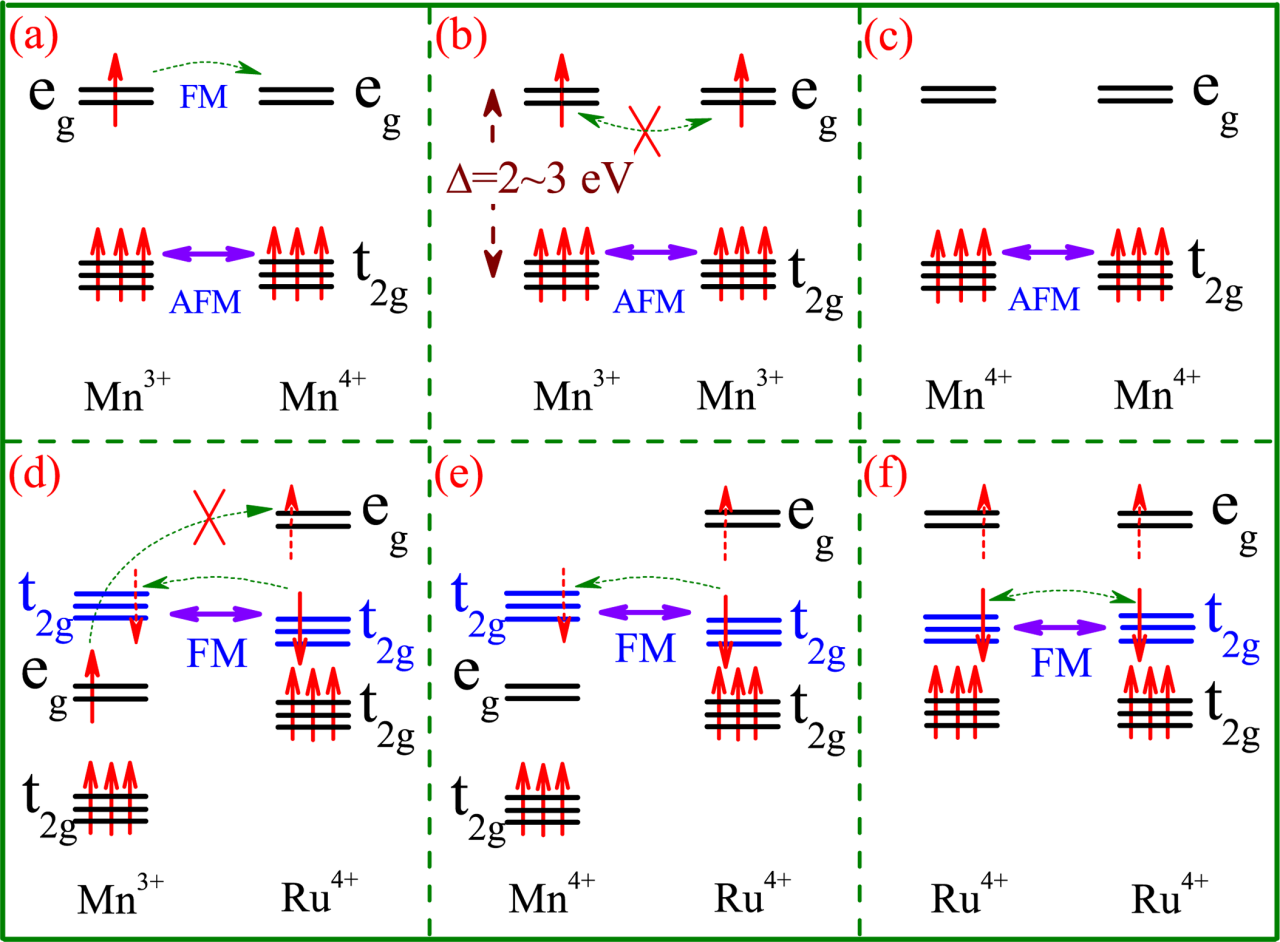
Schematic illustrations of the *e_g_*- and *t_2g_*-orbital alignments between various Me-O-Me pairs where O^2-^ ion is ignored for drawing. (a) Mn^3+^-Mn^4+^ pair, (b) Mn^3+^-Mn^3+^ pair, (c) Mn^4+^-Mn^4+^ pair, (d) Mn^3+^-Ru^4+^ pair, (e) Mn^4+^-Rn^4+^ pair, and (f) Ru^4+^-Ru^4+^ pair. The *e_g_*-*t_2g_* gap Δ for Mn is ~2-3 eV. The dashed (single-head & double-head) arrows indicate the possible electron hopping. The horizontal coarse solid double-head arrows indicate the orbital electron interactions. The vertical red arrows indicate the electron spins. The *e_g_*- and *t_2g_*-orbitals of Ru should be higher than those of Mn respectively.

**Figure 2:**
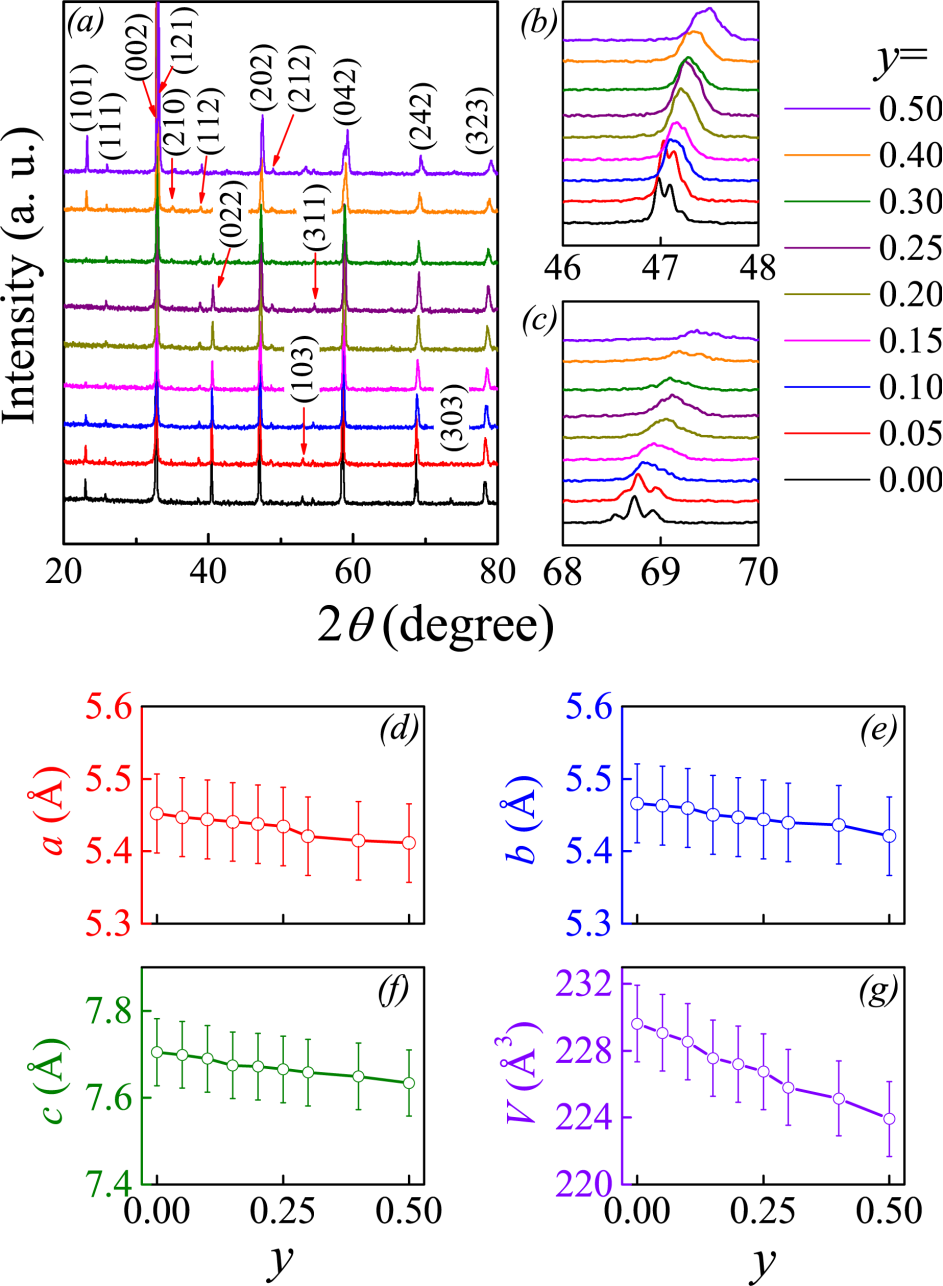
Measured XRD *θ*-2*θ* spectra of a series of samples with different *y* values. (a) The local (202) and (242) reflections are presented in (b) and (c) respectively. The evaluated lattice constants *a*, *b*, *c*, and unit cell volume *V* as a function of *y* are plotted in (d)∼(g) respectively.

**Figure 3:**
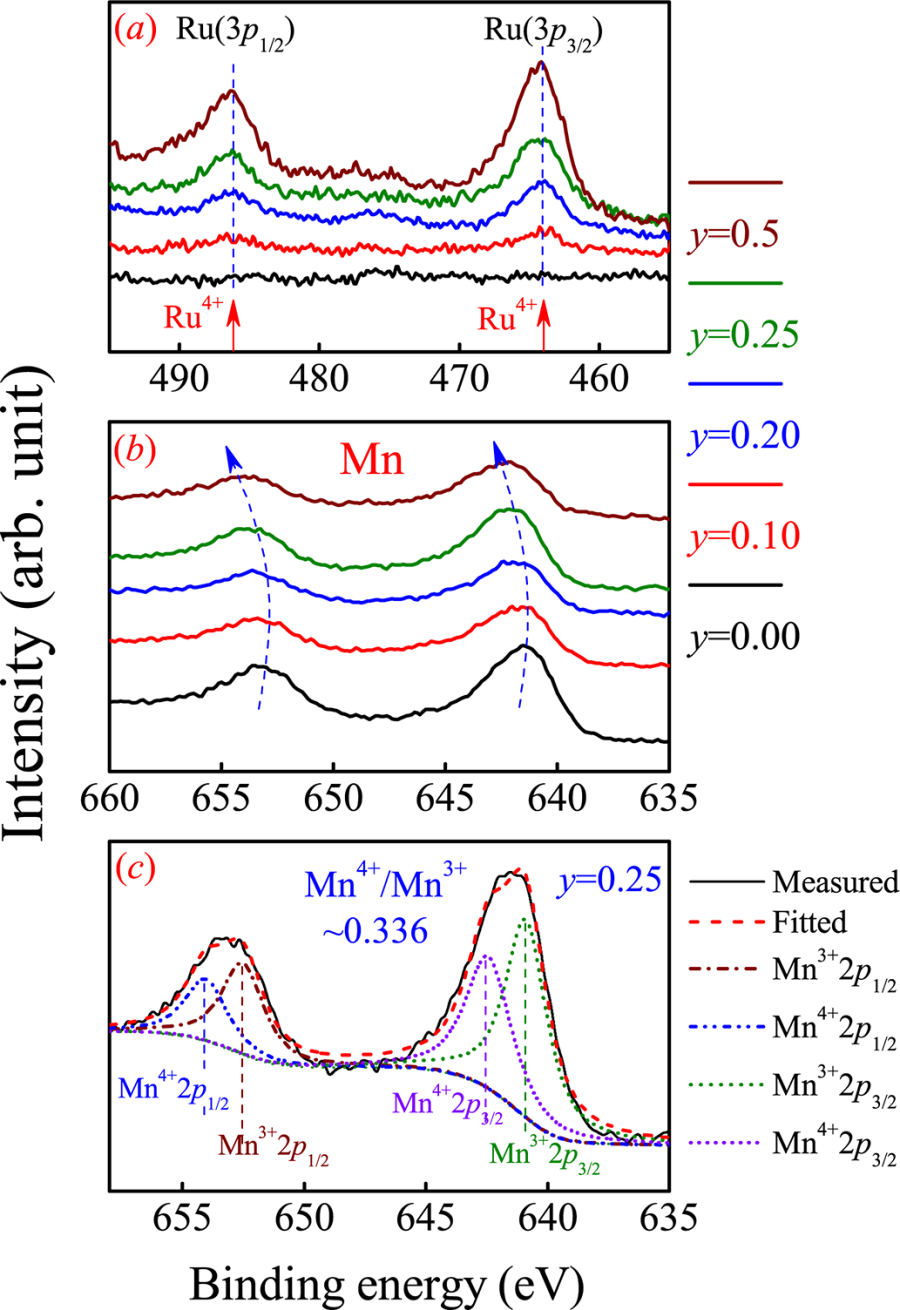
Measured XPS spectra for Ru 3*p*_1/2_ and Ru 3*p*_3/2_ (a), and Mn 2*p*_1/2_ and Mn 2*p*_3/2_ (b) of four samples with different *y*. The Mn^3+^ and Mn^4+^ XPS peak separation fittings for *y* = 0.25 for Mn 2*p*_1/2_ and Mn 2*p*_3/2_ are plotted in (c).

**Figure 4:**
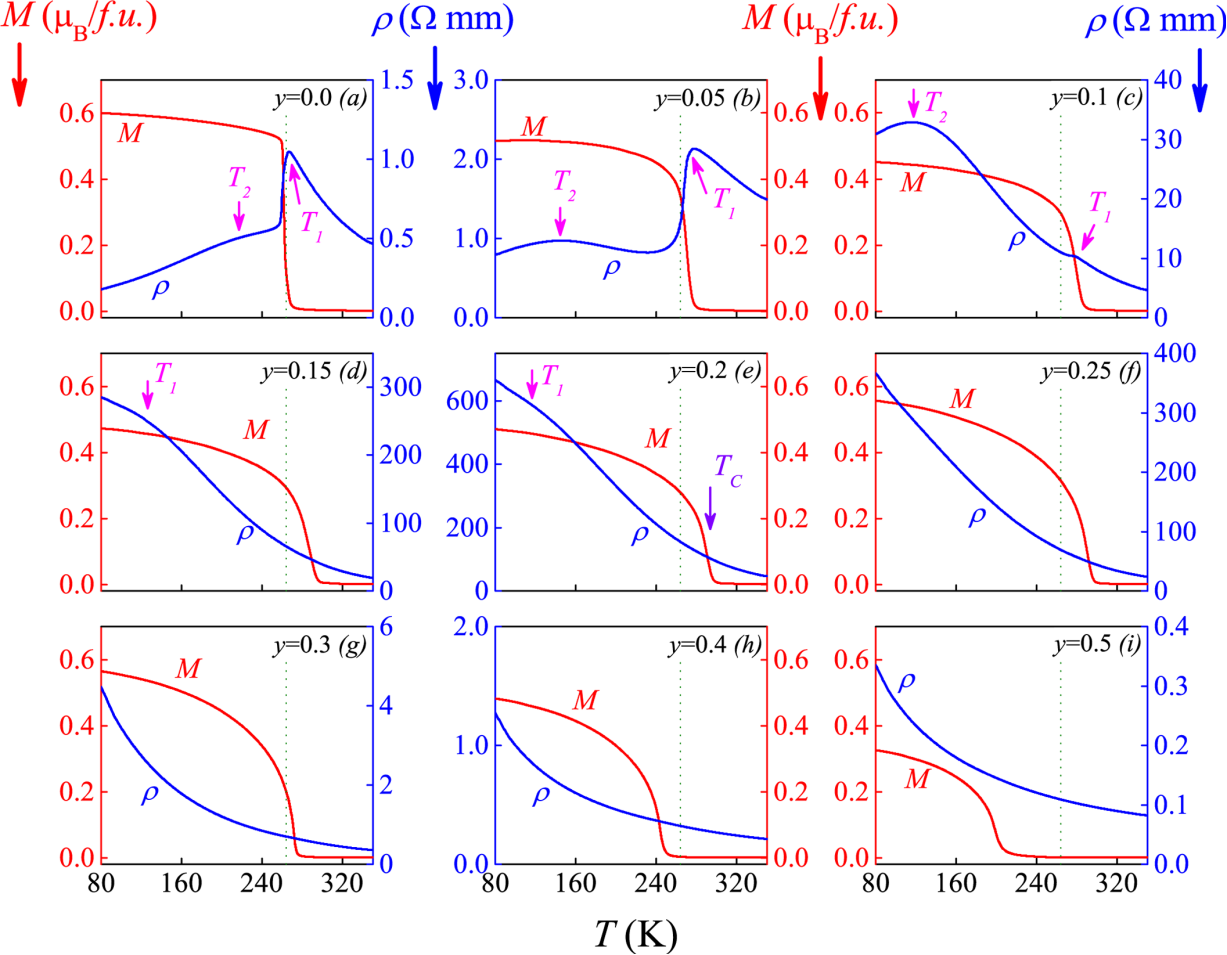
Measured *ρ*(*T*) and *M*(*T*) under field cooling field of 100 Oe for a series of samples. The FM transition point *T_C_* and MIT point *T_1_* are labelled. See text for symbol *T_2_*.

**Figure 5:**
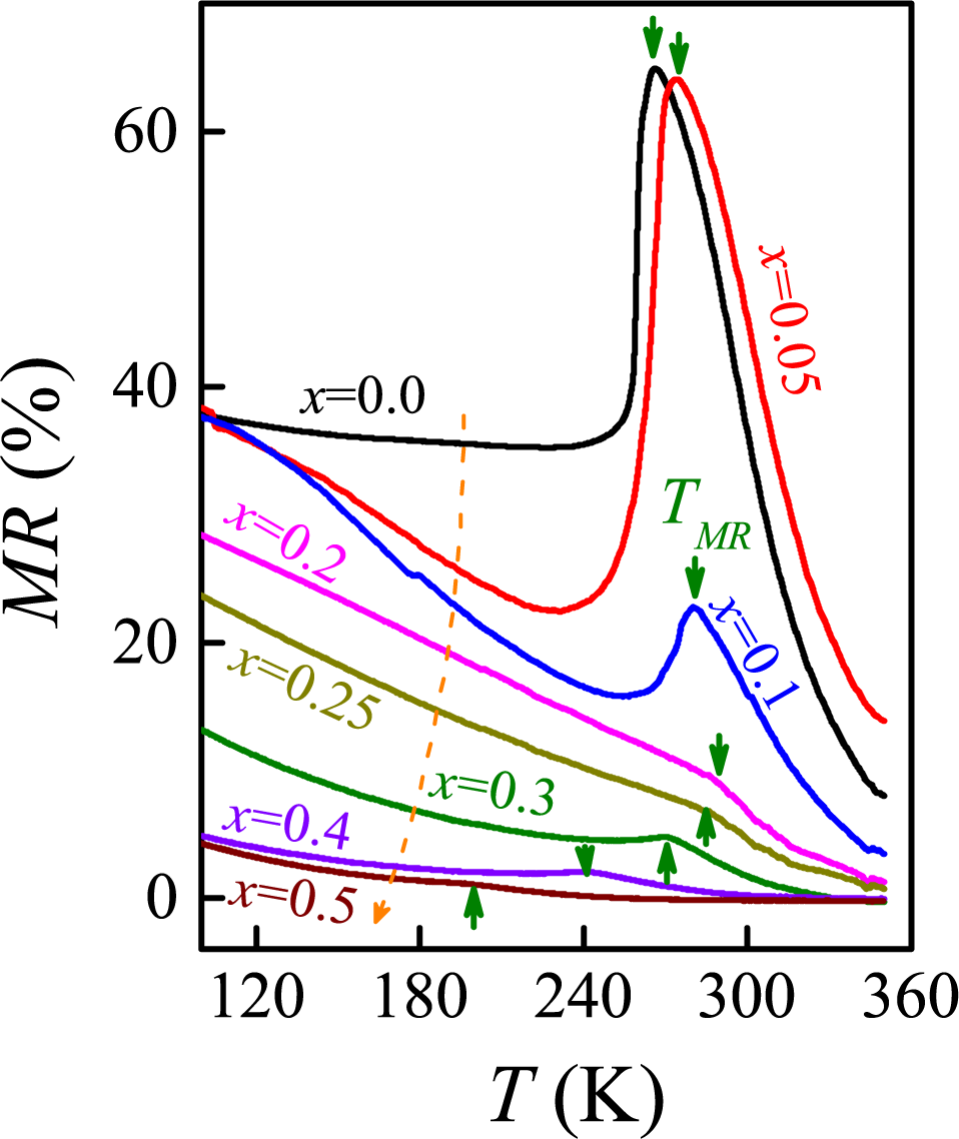
Measured MR(*T*) for a series of samples. The dashed arrows show the *y* sequence (*y* = 0.0, 0.05, 0.10, 0.20, 0.25, 0.30, 0.40, and 0.50). The MR peak point is *T_MR_*.

**Figure 6:**
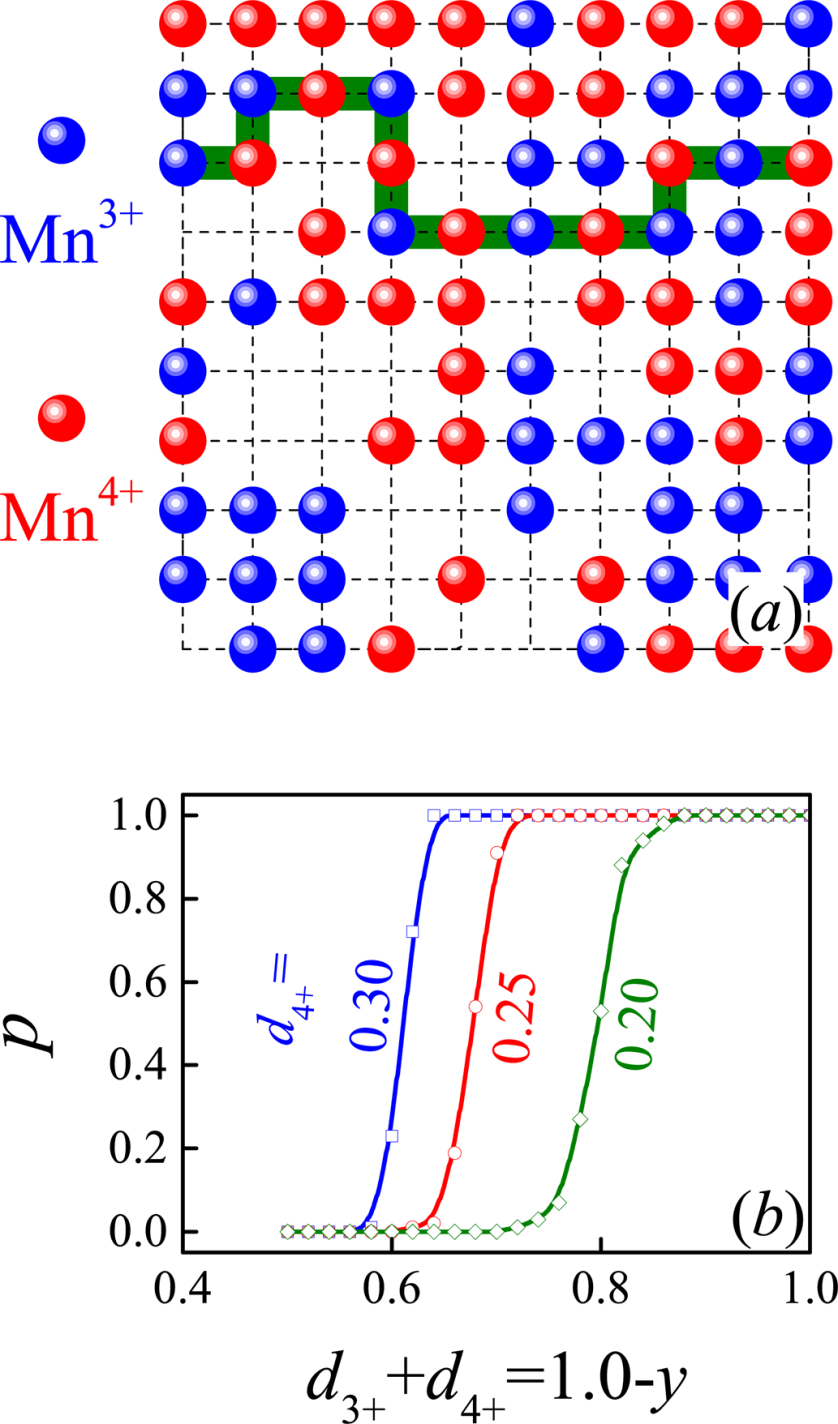
(a) A two-dimensional section of a cubic lattice with random occupation of Mn^3+^ and Mn^4+^, given fixed *d*_3+_ and *d*_4+_. The olive pipe-like channel is conductive due to the alternating Mn^3+^-Mn^4+^ occupation. (b) Simulated conduction probability *p* as a function of (*d*_3+_+*d*_4+_) given three different *d*_4+_ values as indicated. The abrupt change of *p* from 0.0 to 1.0 implies the conduction percolation.

**Figure 7:**
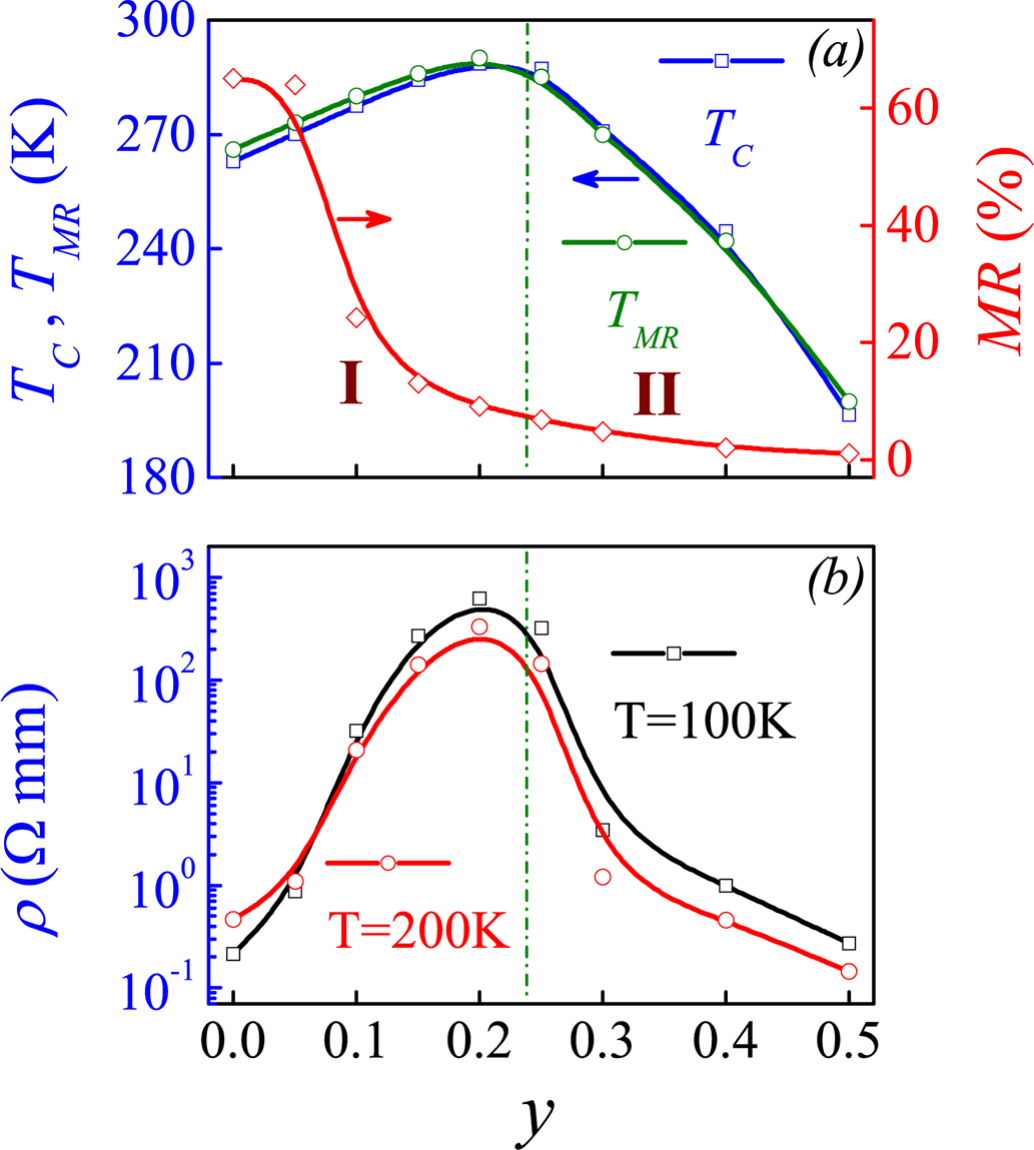
(a) Evaluated FM transition point *T_C_*, MR peak point *T_MR_*, and maximal MR value at *T_MR_* as a function of *y*. (b) Measured resistivity *ρ* at *T* = 100K and 200K as a function of *y* respectively.

**Figure 8:**
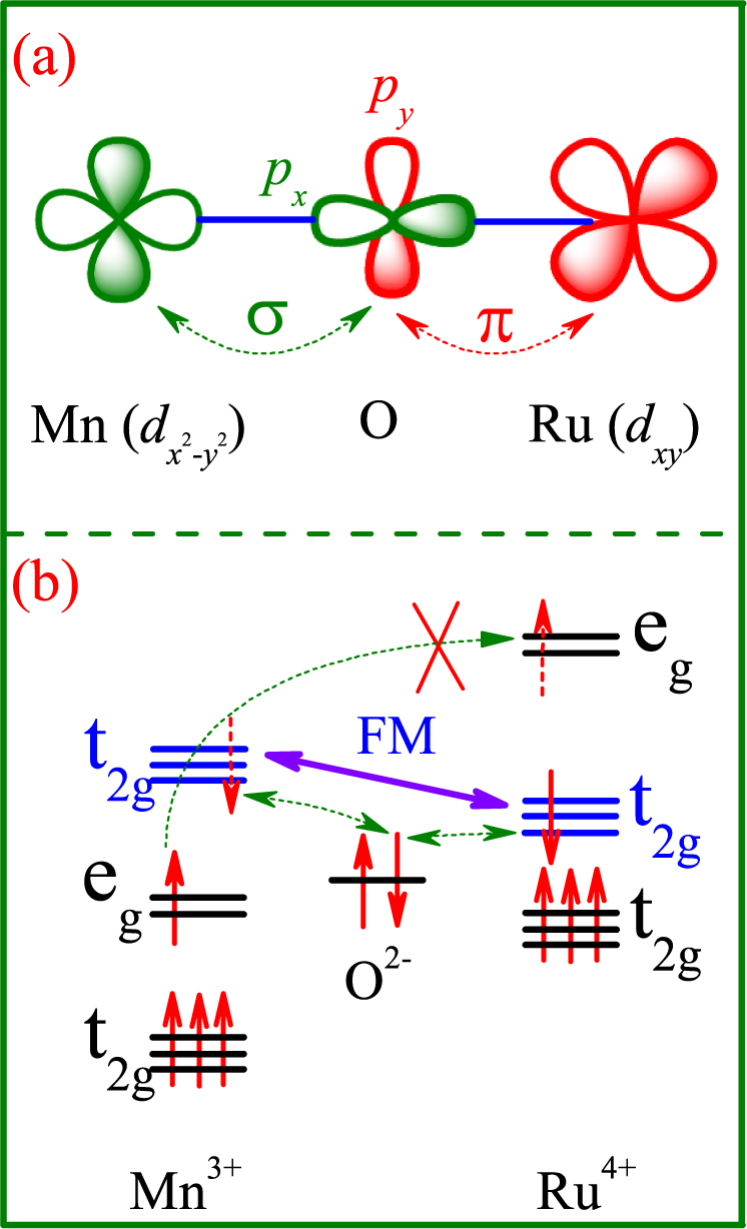
(a) Orbital alignment between Mn 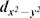-orbital and Ru *d_xy_*-orbital via an intermediate O *p*-orbitals for a Mn^3+^-Ru^4+^ pair, with no interaction between the Mn 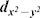-orbital and Ru *d_xy_*-orbital. The olive and white colors represent the opposite charge distributions. (b) The *t_2g_*-orbital alignment of FM interaction for a Mn^3+^-Ru^4+^ pair. The Mn^4+^-Ru^4+^ pair has the similar *t_2g_*-orbital alignment.

To this stage, one can reasonably explain this unusual ferromagnetism enhancement. As shown in Fig. 1(d) and (e), the Mn-Ru FM super-exchange at the *t_2g_*-orbitals is the most probable origin for the ferromagnetism enhancement, while the contribution from the Ru-Ru *t_2g_* FM interaction is negligible in the low-*y* range. It is noted that the *t_2g_* electrons are usually localized^32^. The Mn-Ru FM interactions at the *t_2g_*-orbitals can strengthen the ferromagnetism but have no benefit to electron hopping in the low-*y* range, consistent with our observations. To more clearly illustrate this fact, we have to make sure that the interaction between the Mn *e_g_*-orbitals and Ru *t_2g_*-orbitals if any should be weak. Since the Ru *t_2g_*-orbital levels are relatively higher than the Mn *e_g_*-orbital levels, the Mn *e_g_*-orbitals and Ru *t_2g_*-orbitals may not have an overlap with each other. A schematic of the arrangement of the Mn 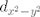-orbital and Ru *d_xy_*-orbital with O 2*p*-orbitals for a Ru^4+^-O-Mn^3+^ bond is given in Fig. 8(a), noting this bond angle is not very far from 180°. It seems that the effective interaction between the Mn 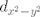 -orbitals and Ru *d_xy_*-orbitals bridged with the O 2*p*-orbitals is not strong since the Mn-O-Ru bond angle is ~180° in the ideal situation^41^. However, it should be mentioned that the Me-O-Me bond angle here (Me = Mn, Ru) is smaller than 180°, probably allowing a weak electron hopping between the Mn^4+^-O^2−^-Ru^4+^ chains. This hopping sequence may contribute somehow to the electrical conduction. In fact, our Rietveld refinements of the XRD data do give a rough estimation of the Mn(2)-O(2)-Ru(1) bond angle to be ~161.71^o^ for sample *y* = 0.05. This angle is already sufficient to avoid the strong interaction between the Mn 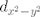 -orbitals and Ru *d_xy_*-orbitals. It is believed that a much smaller angle is needed for such strong interaction. Therefore, what we need to consider is the super-exchange between the Mn *t_2g_*-orbitals and Ru *t_2g_*-orbitals bridged with the O 2*p*-orbitals, as shown in Fig. 8(b). One can immediately predict that the super-exchange between them is FM^29^,^30^ and may be sufficiently strong.

To have a semi-quantitative estimation of this Mn-Ru FM super-exchange, we consult recent experimental data in literature. First, it was reported that for CaMn_1-x_Ru_x_O_3_, a Ru-substitution up to *x* = 0.08 enables the transition of the antiferromagnetic ground state towards the FM state with the transition point *T_C_* as high as ~50K^42^. Similar experiments on CaMn_1-x_Ru_x_O_3_ up to *x*~0.40 revealed a FM state with the transition point *T_C_*~185K while CaMnO_3_ showed an antiferromagnetic state with the Neel point *T_N_*~145K. This suggests that the Mn-Ru FM interaction can be as strong as tens of kelvin^29^. Second, similar Mn-Ru FM effective exchange in manganites can be obtained from the ESR measurements^34^. As mentioned, for *y* = 0, the *d*_3+_ = 0.7 is two times higher than the *d*_4+_ = 0.3, the Mn^3+^-Mn^4+^ double-exchange will not be seriously weakened in the low-*y* range. In the zero-order approximation, the total effective FM exchange may be as big as tens of Kelvin. Therefore, one expects a roughly linear enhancement of *T_C_* with *y* in the low-*y* range^43^, as revealed in our experiments. Surely, this prediction becomes invalid once *y* is sufficiently high, e.g. *y*>0.25 in the present work.

### Quenched disorder and electronic phase separation

**Figure 9:**
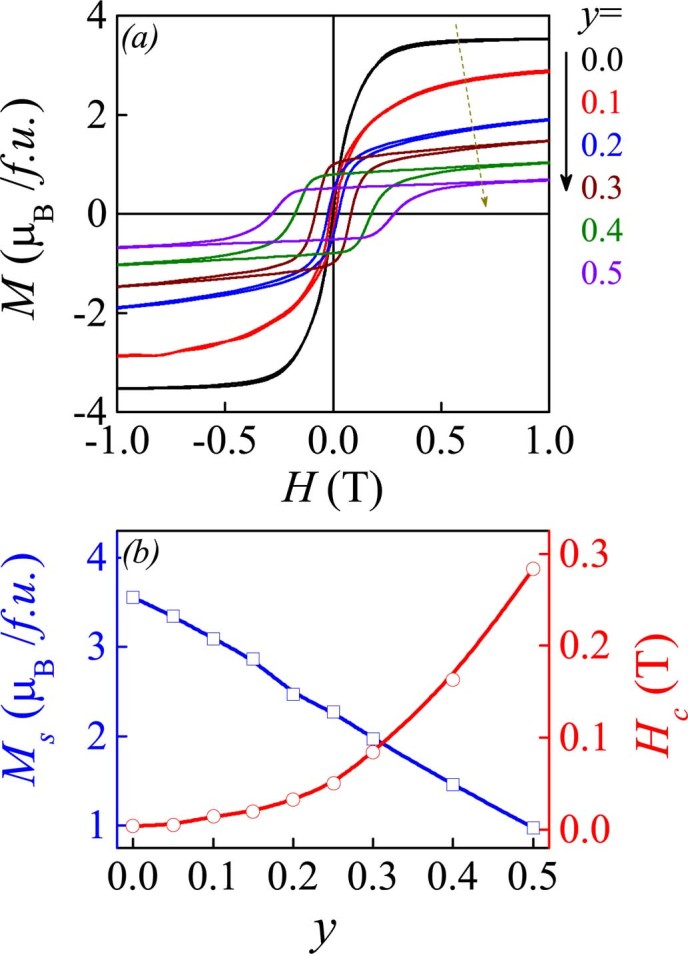
(a) Measured M-H hysteresis loops for several samples at *T* = 2K. (b) Evaluated saturated magnetization *M_s_* and coercive field *H_c_* as a function of *y* at *T* = 2K.

Finally, we discuss the significance of the possible quenched disorder effects associated with the Ru-substitution with synchronous Ca-substitution in LCMRO samples. Anyhow, due to the La^3+^-Ca^2+^ and Mn^3+^-Ru^4+^ ionic size mismatches and charge/orbital fluctuations, the quenched disorder and thus EPS becomes non-negligible^27^. The anomaly of *ρ*(*T*) at *T_2_* is somehow attributed to the EPS. We measured the *M-H* hysteresis loops at *T* = 2K and evaluate the saturated magnetization *M_s_* and coercivity *H_c_* as a function *y*, as shown in Fig. 9(a) and (b). The rapidly increasing *H_c_* (~0.3T at *y* = 0.5) can be used to scale the magnitude of the quenched disorder which pins the magnetic domains from switching, noting that CaRuO_3_ itself is paramagnetic and SrRuO_3_ is ferromagnetic with a coercivity of ~0.1T^32^. At *y*<0.25, the coercivity is sufficiently small, indicating that the disorder effect if any is weak. On the other hand, it is found that the *M_s_* decreases linearly with increasing *y*, suggesting the rapidly suppressed ferromagnetism in the high-*y* range.

## Methods

The LCMRO polycrystalline samples with *x* = 0.3 and *y* = 0.0~0.5 were prepared using the convention solid sintering method in air. The highly purified powder of oxides and carbonates was mixed in stoichiometric ratio, ground, and then fired at 1000°C for 24 h in air. The resultant powder was re-ground and pelletized under a pressure of 1000 psi into disks of 2.0 cm in diameter, and then these pellets were sintered at 1300°C for 24 h in air in prior to natural cooling down to room temperature. The chemical composition and spatial homogeneity were checked using the EDS mapping associated with the scanning electron microscopy (SEM, Ultra 55, Zeiss), confirming quite good spatial homogeneity of La, Ca, Mn, and Ru in the μm scale and nano-scale. The evaluated chemical composition is very close to the nominal one within uncertainty of less than 5%. The crystallinity and structure were checked by X-ray diffraction (XRD) using the Cu *K_α_* radiation at room temperature with an X-ray power diffractometer (D8 advanced, Bruker). The refinement of the XRD data was performed using the Rietveld method. For checking possible valence state fluctuations upon the Ru substitution, the charge states of Mn and Ru ions were examined by X-ray photoelectron spectroscopy (XPS) using a photon energy of 1253.6 eV (Mg Ka).

We carefully measured the electrical resistivity *ρ* and dc magnetization *M* of the as-prepared samples. The *M* as a function of temperature (*T*) and magnetic field (*H*) was measured using the Quantum Design superconducting quantum interference device magnetometer (SQUID) in the zero-field cooled (ZFC) and field-cooling (FC) modes respectively. The cooling field and measuring field were both 100 Oe, sufficiently low so that the magnetic field driven side-effects if any are as weak as possible. In addition, the quasi-static *M*-*H* hysteresis loops under high field at different *T* were measured so that the Ru substitution induced disorder effect can be qualitatively evaluated by characterizing the coercive field (*H_c_*) as a function of the substitution level *y*. The *ρ*(T) and *ρ*(*H*) were measured by a physical properties measurement system (PPMS) from the Cryogenic Co. Ltd and PPMS from the Quantum Design Inc. Since the Mn^3+^-Mn^4+^ double-exchange transport mechanism leads to significant CMR effect occurring around the FM transition point (*T_C_*), we also measured the magnetoresistance MR = [*ρ*(0)-*ρ*(*H*)]/*ρ*(0). The peak of MR(*T*) around the *T_C_* seems to be a symbol for the significance of the double-exchange transport^4^.

## Author Contributions

J.M.L. conceived the research project and M.F.L. performed the measurements. Z.Z.D. and Y.L.X contributed the theoretical analysis. Y.L.X, X.L, Z.B.Y. discussed and commented the results. M.F.L and J.M.L. wrote the manuscript.

## Supplementary Material

Supplementary Information Supplementary Information
