# Supplementary material for: Unusual ferromagnetism enhancement in ferromagnetically optimal manganite La0.7−yCa0.3+yMn1−yRuyO3 (0≤y<0.3): the role of Mn-Ru t2g super-exchange
Source: Sci Rep. 2015 Apr 24;5:9922. doi: 10.1038/srep09922 (PMC4408983; doi:10.1038/srep09922)
Supplement: Supplementary Information [file srep09922-s1.pdf]

Supplementary materials to Scientific Reports

Unusual ferromagnetism enhancement in ferromagnetically optimal manganite $La_{0.7-y}Ca_{0.3+y}Mn_{1-y}Ru_yO_3$ ($0 \le y < 0.3$): the role of Mn-Ru t_{2g} super-exchange

M. F. Liu[1], Z. Z. Du[1], Y. L. Xie[1], X. Li[1], Z. B. Yan[1], and J. –M. Liu[1, 2]

[1]*Laboratory of Solid State Microstructures and Innovative Center of Advanced Microstructures, Nanjing University, Nanjing 210093, China*

[2]*Institute for Advanced Materials and Laboratory of Quantum Engineering and Materials, South China Normal University, Guangzhou 510006, China*

1. Compositional and structural characterizations

In this section, we present the measured data on the spatial distribution of cation ions in the samples. We employed the EDS plane-mapping mode to check the spatial composition homogeneity. The data for sample $La_{0.4}Ca_{0.6}Mn_{0.7}Ru_{0.3}O_3$ (y=0.3) are plotted in SFig. 1. It is seen that the spatial distributions for La, Ca, Mn, and Ru are all homogeneous in the sub-μm scale and nano-scale.

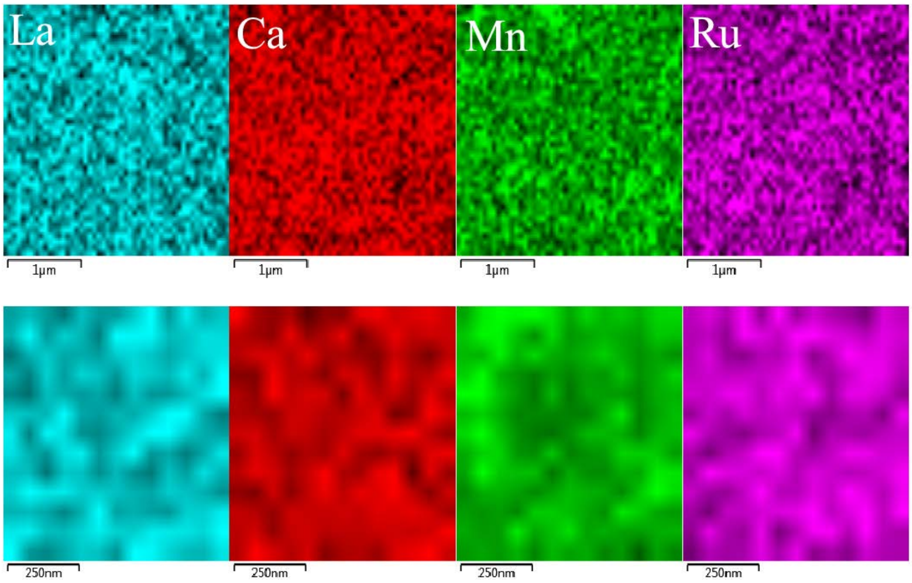

SFig. 1. The plane EDS maps of four species La, Ca, Mn, and Ru, respectively, at two different scales, for

sample $La_{0.4}Ca_{0.6}Mn_{0.7}Ru_{0.3}O_3$ (y=0.3). The scale bars on the top row and bottom row are 1.0 μm and 250 nm, respectively.

At the same time, we also performed Rietveld refinement processing on samples by choosing different CIF data bases to check the cation ratios. Here we take sample $La_{0.65}Ca_{0.35}Mn_{0.95}Ru_{0.05}O_3$ as an example (i.e. y=0.05). The Rietveld refinement data are plotted in SFig. 2 below. It is clearly seen that the Rietveld refinement reliability if one takes the CIF data base for y=0.05 is much higher than that if the CIF data base for y=0.0 are used. This confirms that the cation ratios of the real sample are close to the nominal structure. Similar refinements have been done for several other samples and the conclusion is the same.

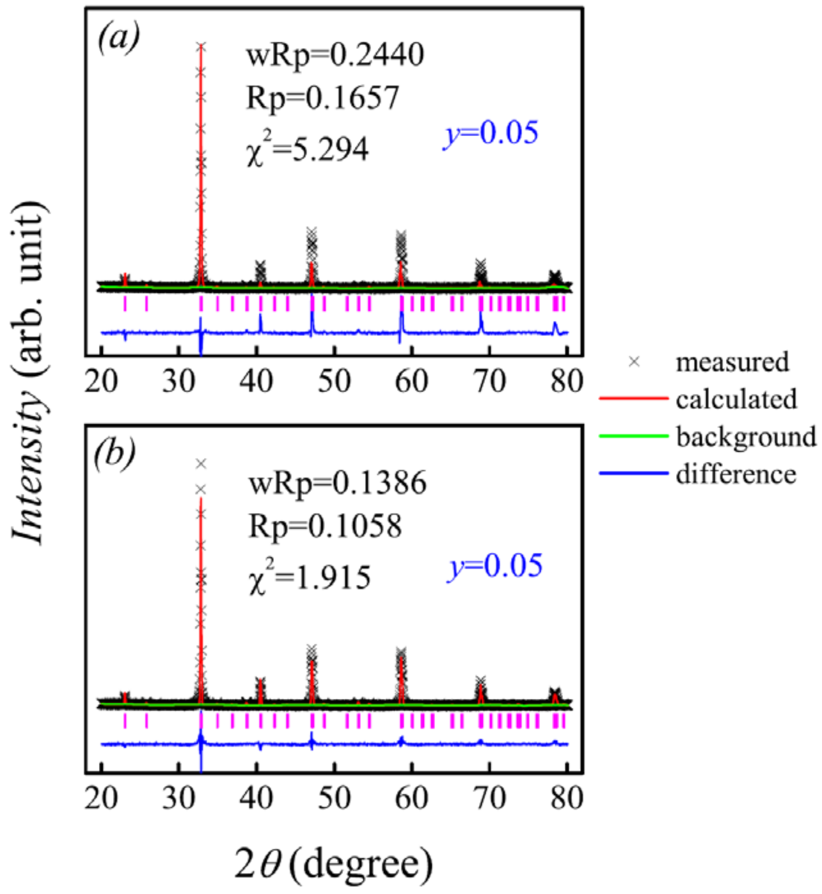

SFig. 2. The Rietveld refined XRD data for sample $La_{0.65}Ca_{0.35}Mn_{0.95}Ru_{0.05}O_3$ (y=0.05). The reference data basis (CIF file) used for the refinements are for $La_{0.7}Ca_{0.3}MnO_3$ (a) and $La_{0.65}Ca_{0.35}Mn_{0.95}Ru_{0.05}O_3$ (b) respectively.

2. Evidence for weak electronic phase separation

In our measurements, we always use the cooling-warming cycle measurements of the *M*-*T* and ρ-*T* curves to justify whether significant phase separation exists in these samples or not. As exampled in the SFig. 3, the almost overlapped *M*-*T* and ρ-*T* curves measured in the cooling and warming sequences indicate no remarkable phase separation over the whole *T*-range. Such a phase separation would be expected to generate remarkable thermal hysteresis at least in the low-*T* range. In addition, no specific anomaly of *M*-*T* and ρ-*T* curves below 90K (including nearly 43 K) can be seen. This excludes possible existence of Mn_3O_4 in the samples. Therefore, one may conclude that such phase separation if any is not dominant in our samples.

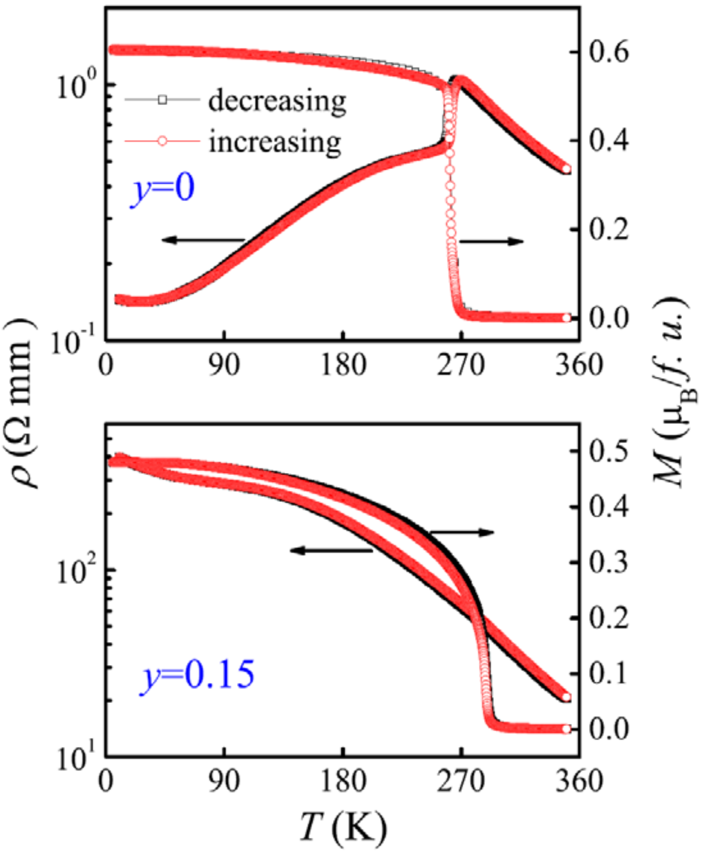

SFig. 3. Measured *M*-*T* and ρ-*T* curves over the whole *T*-range in the cooling-warming cycle for $La_{0.7}Ca_{0.3}MnO_3$ (*y*=0) and $La_{0.55}Ca_{0.45}Mn_{0.85}Ru_{0.15}O_3$ (*y*=0.15). It is seen that the curves measured in the cooling and warming paths are almost overlapped over the whole *T*-range.

In addition, we measured the ρ-*T* and *M*-*T* curves over 2 K~350 K for all the samples

with y≤0.5. Here, T_2 marks the weak charge-ordering as identified from the ρ-T curves.

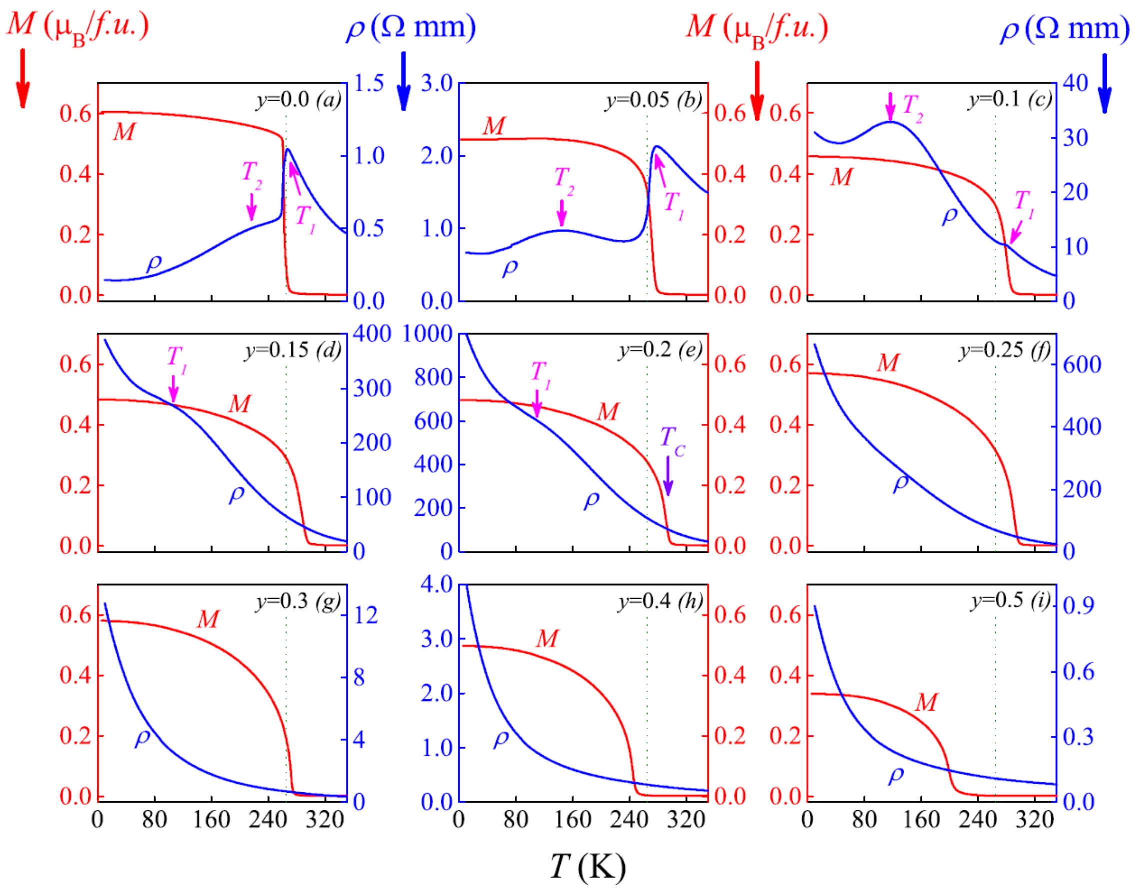

SFig. 4. Re-plotting the M-T and ρ-T data shown in Fig. 4 of the manuscript. Here the covered T-range is from 2K to 350K. No MIT transition can be seen at x>=0.25 over the whole T-range.
